# Exon Expression Arrays as a Tool to Identify New Cancer Genes

**DOI:** 10.1371/journal.pone.0003007

**Published:** 2008-08-20

**Authors:** Mieke Schutte, Fons Elstrodt, Linda B. C. Bralten, Jord H. A. Nagel, Elza Duijm, Antoinette Hollestelle, Maartje J. Vuerhard, Marijke Wasielewski, Justine K. Peeters, Peter van der Spek, Peter A. Sillevis Smitt, Pim J. French

**Affiliations:** 1 Department of Medical Oncology, Josephine Nefkens Institute, Erasmus University Medical Center, Rotterdam, The Netherlands; 2 Department of Neurology, Josephine Nefkens Institute, Erasmus University Medical Center, Rotterdam, The Netherlands; 3 Department of Bioinformatics, Josephine Nefkens Institute, Erasmus University Medical Center, Rotterdam, The Netherlands; Sanofi-Aventis, United States of America

## Abstract

**Background:**

Identification of genes that are causally implicated in oncogenesis is a major goal in cancer research. An estimated 10–20% of cancer-related gene mutations result in skipping of one or more exons in the encoded transcripts. Here we report on a strategy to screen in a global fashion for such exon-skipping events using PAttern based Correlation (PAC). The PAC algorithm has been used previously to identify differentially expressed splice variants between two predefined subgroups. As genetic changes in cancer are sample specific, we tested the ability of PAC to identify aberrantly expressed exons in single samples.

**Principal Findings:**

As a proof-of-principle, we tested the PAC strategy on human cancer samples of which the complete coding sequence of eight cancer genes had been screened for mutations. PAC detected all seven exon-skipping mutants among 12 cancer cell lines. PAC also identified exon-skipping mutants in clinical cancer specimens although detection was compromised due to heterogeneous (wild-type) transcript expression. PAC reduced the number of candidate genes/exons for subsequent mutational analysis by two to three orders of magnitude and had a substantial true positive rate. Importantly, of 112 randomly selected outlier exons, sequence analysis identified two novel exon skipping events, two novel base changes and 21 previously reported base changes (SNPs).

**Conclusions:**

The ability of PAC to enrich for mutated transcripts and to identify known and novel genetic changes confirms its suitability as a strategy to identify candidate cancer genes.

## Introduction

Cancer is driven by mutations in genes that control the proliferation of cells, their survival and their integrity. Screens aimed at identifying such cancer genes often use chromosomal location and/or functional properties to select candidates genes for subsequent mutation analysis [1]–[4]. Although many candidate cancer gene loci have been identified, the labor-intensive mutation analysis severely hampers finding the corresponding cancer gene. Other gene search strategies have focused on aberrant gene expression patterns to identify candidates. For example, gene mutants that result in premature termination codons were identified by screening for genes that were specifically expressed following chemical inhibition of nonsense mediated RNA decay [5]. Furthermore, fusion genes in prostate cancer were identified by screening for outliers in a large cohort of gene-expression profiles [6].

Human cancer gene mutations frequently result in the skipping of one or several exons from the encoded transcripts [7]–[9]. Exon-skipping mutations may be caused by nucleotide substitutions within the consensus splice sites or by deletions that span entire exons. In addition, exon-skipping mutations may be caused by relatively small intragenic insertions, deletions or duplications. Even though exon-skipping mutations represent an estimated 10–20% of all cancer-related gene mutations [4], [9]–[12], no high throughput method has been available to screen for such mutations. Here, we describe Pattern Based Correlation (PAC) as an approach to identify candidate cancer genes by screening for exon-skipping events in a global fashion. Detailed mutation analysis is then restricted only to the PAC-identified outlier exons. As a proof-of-principle, we demonstrate the efficacy of the PAC strategy on previously identified exon-skipping mutations in breast cancer cell lines and in clinical brain tumor samples. We also demonstrate that PAC can identify novel exon skipping events with underlying genetic changes in known cancer genes and in randomly-selected PAC-identified outlier exons.

## Results

### Outlier exon identification by Pattern Based Correlation (PAC)

**Figure 1:**
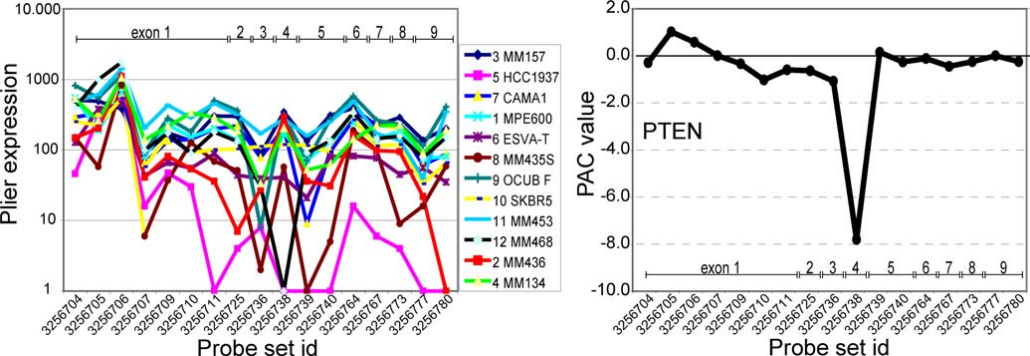
PAC detection of an exon-skipping *PTEN* mutant. (A) Normalized expression data of all exons within the *PTEN* gene. Each exon probe set is represented by a dot in the solid line; multiple probe sets may be directed against the same exon. (B) PAC normalizes the variability in gene expression levels between samples and, in a single sample, the variability in signal intensity between probe sets of the same transcript. PAC calculation therefore allows rapid detection of skipping of *PTEN* exon 4 in breast cancer cell line MDA-MB-468 due to a *PTEN* c.253+1G>T splice site mutation that we previously had identified [17].

In this study we have developed a new approach to screen exon-skipping events in human cancer samples. Because mutations in cancer often are highly heterogeneous with respect to their intragenic location, individual tumors often express unique RNA species. Screening for mutations that result in skipping of one or more exons in the encoded transcript therefore requires screening for unique, exon-skipped, transcripts within a specific sample cohort. Briefly, exon-level expression profiles are generated using Affymetrix Human Exon Arrays, which determine the expression level of virtually all exons present in the human genome. The PAttern based Correlation (PAC) algorithm is then used to calculate the predicted expression level of each exon (or probe set). We then identify outlier exons by subtracting the predicted expression level of exons from their measured expression level, with values equaling zero when the measured expression level of an exon was similar to its predicted expression level (formulated in detail under Methods). The PAC algorithm has been used to identify differential splicing between predefined groups [13]. In this study, we have tested the PAC algorithm for its ability to identify aberrantly expressed exons in single samples of a well defined cohort of cell lines or tumors. PAC effectively normalizes the variability in gene expression levels between samples and, in a single sample, normalizes the variability in signal intensity between probe sets of the same transcript (Fig. 1).

### PAC detects exon-skipping events in breast cancer cell lines

We tested the feasibility of the PAC strategy on a panel of 12 human breast cancer cell lines that had been screened for mutations in seven tumor suppressor genes: *BRCA1*, *CDH1*, *MAP2K4*, *PTEN*, *p16*, *p53* and *RB1* [14]–[18], and unpublished results). Mutation analysis was performed by sequencing of the complete coding sequences of the genes and analysis of all mutations on both genomic gene fragments and transcripts. Together, the 12 cell lines contained seven gene mutants that should be detectable by PAC, as they resulted in the skipping of eight exons from among four tumor suppressor genes (mutations are detailed in Table 1). We have explored the PAC strategy at different cut-off levels, identifying outlier exons that were expressed less than 16-fold, 8-fold, 4-fold, 2.8-fold and 2.5-fold than their predicted expression level (i.e. PAC values of -4.0, -3.0, -2.0, -1.5 and -1.3, respectively). Outlier exons were identified without prior knowledge of the mutation data.

**Table 1:** Detection of exon-skipping mutants by PAC.

Tumor sample	Gene	Mutation	mRNA	PAC detection
OCUB-F	*CDH1*	c.49_163del	r.49_163del115 (Ex2)	detected
MDA-MB-134VI	*CDH1*	c.688_832del	r.688_832del145 (Ex6)	detected
MPE600	*CDH1*	c.1138_1320del	r.1138_1320del183 (Ex9)	detected
CAMA-1	*CDH1*	c.1712-1G>A	r.1566_1712del147 (Ex11)	detected
MDA-MB-468	*PTEN*	c.253+1G>T	r.210_253del44 (Ex4)	detected
MDA-MB-453	*p53*	c.994_1182del	r.994_1182del189 (Ex10-11)	detected
HCC1937	*RB1*	c.2212_2325del	r.2212_2325del114 (Ex22)	detected
Glioblastoma 67	*EGFR*	c.89_889del	r.89_889del801 (Ex2-7)	not detected
Glioblastoma 96	*EGFR*	c.89_889del	r.89_889del801 (Ex2-7)	detected
Glioblastoma 142	*EGFR*	c.89_889del	r.89_889del801 (Ex2-7)	detected
Glioblastoma 149	*EGFR*	c.89_889del	r.89_889del801 (Ex2-7)	not detected
Glioblastoma 163	*EGFR*	c.89_889del	r.89_889del801 (Ex2-7)	not detected
Glioblastoma 164	*EGFR*	c.89_889del	r.89_889del801 (Ex2-7)	not detected

Stated are mutations that result in the expression of an aberrant transcript variant in breast cancer cell lines (top) and clinical glioblastoma samples (bottom). All mutations were determined by sequencing genomic DNA fragments as well as transcripts, where the cDNA sequence c.1 corresponds to the adenosine residue of the ATG initiation codon in the Genbank reference sequence. Genbank accession numbers: Z13009, NM_000314, NM_000546, NM_000321 and NM_005228.3 for *CDH1*, *PTEN*, *p53*, *RB1* and *EGFR*, respectively. Detection of the mutations by PAC was at PAC value −1.3.

**Figure 2:**
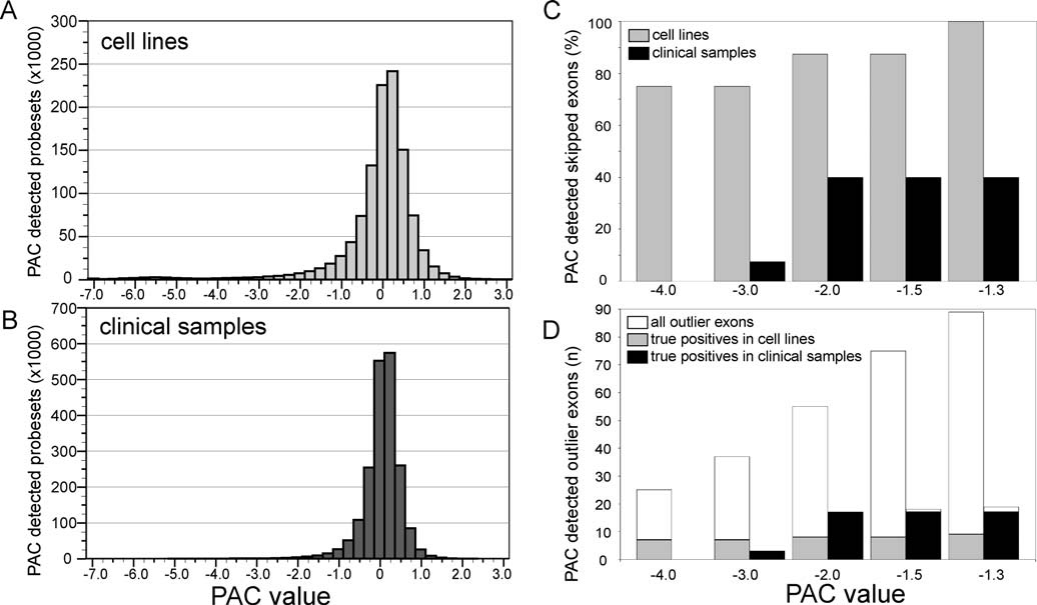
Performance of PAC to detect exon-skipping mutants. (A) and (B) Total number of PAC-detected outlier probe sets from among 290,000 core probe sets in 12 breast cancer cell lines and in 14 glioblastomas, respectively. (C) Number of skipped exons detected by PAC as a percentage of all eight skipped exons present in the breast cancer cell lines, or as a percentage of the 36 skipped *EGFR* exons present in the glioblastomas (see Table 1). (D) Total number of outlier exons (true plus false positives) and number of true positive outlier exons detected by PAC among the seven tumor suppressor genes and the *EGFR* oncogene. True positive outlier exons include all PAC detected skipped exons and two missense mutations (*PTEN* c.274G>C in CAMA1, *MAP2K4* c.551C>G in MDA-MB-134VI).

From the total of 3.4 million core probe sets that we assayed for the 12 cell lines (290,000 core probe sets per sample), PAC identified 21,151 (0.6%) outlier probe sets at PAC value −4.0 and 94,590 (2.8%) outlier probe sets at PAC value −1.3 (Fig. 2A). All probe sets at PAC values <−2.0 (34,137 probe sets corresponding to 31,357 exons and 10,247 genes) are listed in supplementary data Table S1. When all PAC values are plotted in a frequency histogram, a tail towards the negative end is observed (Fig. 2A). This skewed distribution gives a rough estimation of the false positive rate at the various PAC levels. PAC of the seven fully characterized tumor suppressor genes in the 12 cell lines involved analysis of 1,200 exons (1,752 probe sets). PAC correctly detected six of the eight skipped exons when using PAC value −4.0, seven skipped exons were detected at PAC value −2.0 and all eight skipped exons were detected at PAC value −1.3 (Fig. 2C). Importantly, the number of false positive outlier exons was substantially reduced at PAC value −4.0 as compared to PAC value −1.3, resulting in an increase of the true positive rate from 9% to 24% of the identified outlier exons (Fig. 2D). For comparison, random sampling of 24/1200 exons has a >85% probability of not finding any true positive mutation and only a <10^−8^ chance of finding 6 or more. For the known cancer genes used in our study, the true positive rate of our approach thus by far exceeds random exon selection. In this respect, the reduction of the number of false positive candidate genes may initially be far more beneficial for a gene search project than accurate identification of all true positive outlier exons. Together, our results show that the PAC strategy is reliable in detecting exon-skipping mutants in cancer cell lines.

### PAC performance in samples with heterogeneous transcript expression

**Figure 3:**
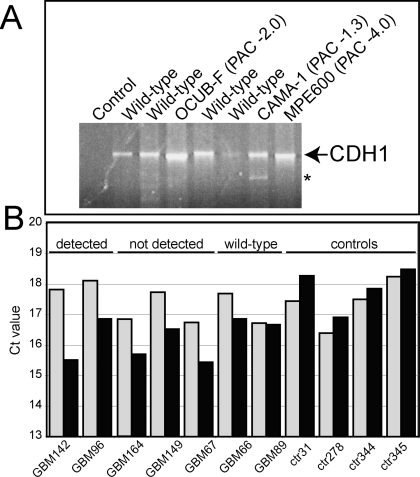
Compromised detection due to heterogeneous transcript expression. Skipping of *CDH1* exon 11 in breast cancer cell line CAMA-1 was only detected at PAC value −1.3, likely due to expression of a second aberrant transcript variant (*) that was detected by conventional RT-PCR. (B) Expression of *EGFR* transcripts was detected in glioblastoma samples by Real-Time RT-PCR, using primers designed to anneal inside the exon 2–7 deletion region of the *EGFRvIII* isoform (gray bars) or outside the deletion region (black bars). Differences in Ct values between the two transcript fragments are indicative for *EGFRvIII* isoform expression levels. All five samples with the *EGFRvIII* isoform also expressed significant amounts of wild-type *EGFR* transcripts, likely compromising outlier detection by PAC (indicated by “detected” and “not detected”). Wild-type, samples with normal transcripts; Controls, non-malignant brain specimens.

Similar to other genetic screening methods, PAC is most suited to detect homozygous genetic changes. For example, the lowest PAC value when 50% wild-type transcripts are present (as may be the case for a heterozygous genetic change) is −1.0. The somewhat compromised detection of skipped exons at PAC value −4.0 as compared to PAC value −1.3 (i.e. six vs. all eight skipped exons) in our panel of breast cancer cell lines therefore may have been caused by the expression of a second aberrant transcript that still includes (part of) the exon. Indeed, a second *CDH1* transcript length of minor intensity was detected in CAMA-1 (Fig. 3A), the splice site mutant that had been detected only at PAC value −1.3.

To further asses the performance of PAC in samples with heterogeneous (wild-type and mutant) transcript expression, we performed PAC on 14 clinical glioblastoma specimens (selected to contain >70% tumor nuclei) that had genomic amplifications of the *EGFR* oncogene. Glioblastomas with *EGFR* amplifications frequently carry an intragenic deletion of exons 2 through 7, resulting in expression of the constitutively active *EGFRvIII* isoform [8], [19]. However, glioblastomas expressing the *EGFRvIII* isoform also frequently express wild-type *EGFR* transcripts. This heterogeneous *EGFR* expression is related to amplification of the *EGFR* locus prior to the deletion of exons [20], although non-malignant cells in the glioblastoma specimens may also express *EGFR*. Of the fourteen glioblastoma samples used in this study, six expressed *EGFRvIII* (a total of 36 skipped exons) of which five also expressed significant levels of wild-type *EGFR* transcripts as determined by quantitative Real-Time PCR (qPCR) (Fig. 3B) (insufficient RNA remained of the sixth sample with *EGFRvIII* expression to perform qPCR).

From the total of 4.1 million core probe sets that we assayed for these 14 samples (290,000 core probe sets per sample), PAC identified 1,646 (0.04%) outlier probe sets at PAC value −4.0 and 39,936 (1.0%) outlier probe sets at PAC value −1.3 (Fig. 2B). PAC thus identified three to ten-fold less outlier exons in the glioblastomas as compared to the breast cancer cell lines (Fig. 2A). All probe sets at PAC values <−2.0 (11,287 probe sets, corresponding to 10,903 exons and 6,264 genes) are listed in supplementary data Table S1. This smaller number of outlier exons in the glioblastomas may be related to their homogeneous histopathology and their highly similar gene expression profiles [13], [21], to the presence of non-neoplastic cells in the tumor samples, or may reflect sampling biases due to small cohort sizes.

PAC of the *EGFR* gene in the 14 glioblastomas involved the analysis of 392 exons (434 probe sets). PAC detected two of six *EGFRvIII* expressing tumors (12 of the 36 skipped exons) at PAC values −2.0 and lower (Table 1 and Fig. 2C). Of the two glioblastomas with *EGFRvIII* that had been detected by PAC, one had significantly (i.e. >5 fold) more mutant than wild-type *EGFR* transcripts. In this tumor, the Ct value difference was >2 between qPCR fragments inside (measuring only wild-type *EGFR* transcripts) and outside (measuring both wild-type and *EGFRvIII* transcripts) the *EGFR* exon 2–7 deletion region (Fig. 3B). The other glioblastoma had a similar expression level difference between wild-type and *EGFRvIII* transcripts (Ct value difference of ∼1.5) as the three glioblastomas that had not been detected by PAC, but had lower overall *EGFR* transcript levels. It appears that PAC detection of the *EGFRvIII* isoform is determined by the overall expression level of *EGFR* transcripts in combination with the ratio of *EGFRvIII* and wild-type *EGFR* transcripts, where samples with too high *EGFR* transcript levels may escape PAC detection due to saturation of the probe sets involved. These results show that the PAC strategy can detect exon-skipping mutants in clinical cancer specimens if the ratio mutant/wild-type transcript level is high and when probe sets are within the linear detection range of the microarray.

### PAC performance in detecting recurrent outlier exons

**Figure 4:**
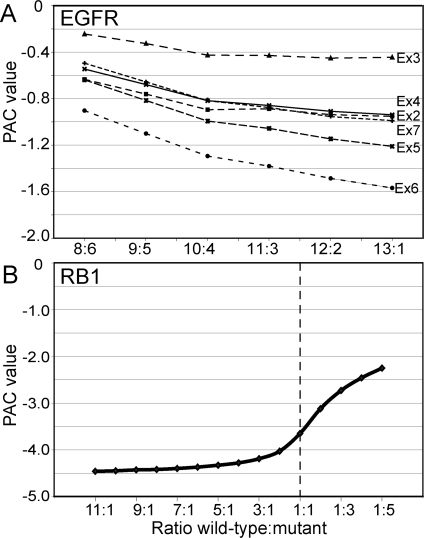
Performance of PAC to detect recurrent outlier exons. (A) Simulation experiment to determine PAC performance in detecting recurrent exon-skipping events among clinical glioblastoma samples, where mutant samples express the *EGFRvIII* isoform with deletion of exons 2 through 7. The cohort of 14 glioblastomas included six mutant samples that were replaced by wild-type samples through reiteration, based on their position from left to right in Fig. 3B. Deletion of *EGFR* exon 6 in sample GBM67 was detected only as unique mutant sample. (B) Simulation experiment to determine PAC performance in detecting recurrent exon-skipping events among breast cancer cell lines, using the wild-type cell line CAMA-1 and the *RB1* exon 22 deletion mutant HCC1937. The two cell lines were analyzed under various cohort sizes, with either the wild-type or the mutant cell line as single sample. The mutant sample was still detected at PAC value −2.0 with five recurrent mutants among six samples. The average expression level of *RB1* exon 22 dropped below PLIER 50 when more than five mutants were simulated, precluding PAC analysis (see Materials and Methods).

PAC performance can also be challenged by recurrent outlier exons. Such frequently skipped exons will result in an underestimation of the exon/transcript ratio in the PAC algorithm and so increase PAC values. We therefore evaluated the performance of PAC in detecting recurrent outlier exons by reiterated replacement of *EGFRvIII* expressing samples with samples that expressed only wild-type *EGFR* (Fig. 4A). When six of 14 samples express *EGFRvIII*, the deletion of exons 2–7 in GBM67 is not detected by PAC. PAC values indeed decreased with decreasing ratios of wild-type versus mutant samples. However, the decrease was relatively small and resulted in the identification of only one of the six deleted exons once the ratio had dropped to one mutant sample among 14 samples. We also simulated PAC detection of recurrent mutations with two breast cancer cell lines, of which HCC1937 had skipped *RB1* exon 22, and we were already able to identify the mutant from among two samples up to even five mutants from among six samples (Fig. 4B). These simulation experiments indicate that PAC performs well in identifying recurrent exon-skipping mutations.

### Detection of nucleotide substitutions and novel genetic changes by PAC

The performance of PAC was further evaluated by analysis of outlier exons selected from all candidates at PAC value ≤−2.0 in breast cancer cell lines and clinical glioblastoma samples. In total, 44 and 68 outlier exons were screened in breast cancer cell lines and glioblastoma samples respectively. Sequence analysis of PCR amplified outlier exons identified two novel exon skipping events and two novel genetic base changes in glioblastoma samples, as well as a number of previously reported base changes (homozygous SNPs) in breast cancer cell lines (n = 5) and glioblastomas (n = 16). RT-PCR experiment results are detailed in supplementary data Table S2.

**Figure 5:**
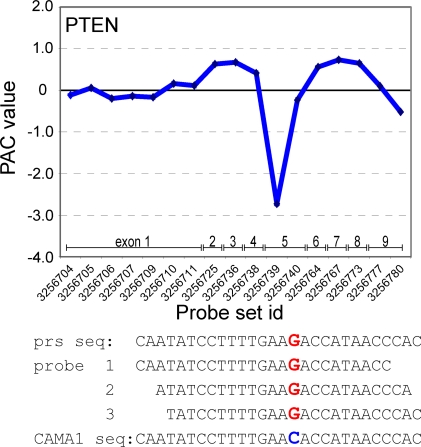
Identification of nucleotide substitutions by PAC. (A) PAC predicts skipping of the 5′ end of *PTEN* exon 5 in the CAMA-1 breast cancer cell line. This cell line contains a nucleotide substitution within the identified exon. This base change does not induce exon skipping but is centrally located within all three probes of the probe set (B). The central location suggests this mutation causes a reduced affinity to the probes on the exon-array.

The majority of genetic changes identified by PAC were single nucleotide changes, both in breast cancer cell lines (five known SNPs) and in glioblastomas (two novel base changes and 16 known SNPs). Moreover, two out of ten previously identified oncogenic point mutations that did not result in exon skipping events were also PAC detected in our cohort of breast cancer cell lines: *MAP2K4* c.551C>G in MDA-MB-134VI and *PTEN* c.274G>C in CAMA-1; [16], [17] (Fig. 5). Single nucleotide mismatches have been used to define hybridization specificity on other Affymetrix microarray platforms. By analogy, single nucleotide substitutions in cancer may also cause reduced hybridization to the probes on the microarray and thus be detected as outlier exons by PAC. Indeed, all of the PAC detected base changes and SNPs were centrally localized within the probe set selection region and overlap with several of its individual probes (Fig. 5).

**Figure 6:**
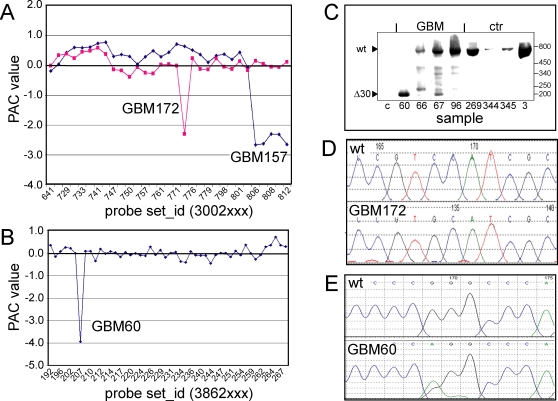
PAC Identification of novel genetic changes. (A) PAC detection of novel genetic changes in *EGFR*. PAC predicted skipping of the last four exons of GBM157 and the 5′ end of exon 17 in GBM172. Semi-quantitative PCR on genomic DNA confirmed the deletion in GBM157 (not shown). (B) PAC predicts skipping of exon 30 in the *FCGBP* gene in GBM60. (C) RT-PCR confirmed the *FCGBP* exon skipping event in GBM60; other tumors did not show this exon skipping. (D) Direct sequencing identified a single base change in *EGFR* in GBM172 (as predicted by PAC, see Fig. 6A). (E) Confirmation of a PAC predicted change in the *TLE2* gene in GBM60. The nucleotide substitution overlaps with individual probes of the probe set.

One of the PAC identified novel exon skipping events was predicted to result in a deletion of the four 3′ end exons of *EGFR* (Fig. 6A). This exon-skipping event was due to a genomic deletion as determined using semi quantitative PCR on genomic tumor DNA. Compared to the 5′ end of the *EGFR* locus in GBM157, the 3′ end showed less amplification (ΔCt −2.5) whereas other samples showed equal amplification between the 5′ and 3′ end of the gene (ΔCt 0.3±1.9). Similar 3′ deletions in *EGFR* have been observed previously in gliomas [19]. The second exon-skipping event predicted by PAC would result in a deletion of exon 30 in the *FCGBP* cDNA (Fig. 6B). This deletion will cause a frameshift that is predicted to result in a truncated protein. The absence of exon 30 was confirmed by RT-PCR and sequence analysis (Fig. 6C). Novel identified single base changes include a single base change 1934C>G (S645C) in the *EGFR* gene, (Fig. 6A and D), and a single base change 946G>A (G316R) in the *TLE2* gene (Fig. 6E). The G316R (946G>A) mutation in *TLE2* is rendered “pathological” by PMut (mmb2.pcb.ub.es:8080/PMut/) and “not tolerated” by SIFT BLink (blocks.fhcrc.org/sift/SIFT_BLink_submit.html). In summary, the novel exon skipping events and base changes identified by analysis of a selected set of outlier exons confirms the suitability of PAC to identify candidate cancer genes.

## Discussion

We have developed an approach that uses PAttern based Correlation (PAC) to screen for cancer gene mutations that cause exon skipping in the encoded transcripts. We demonstrate that PAC correctly detected all of seven previously identified exon-skipping mutants in breast cancer cell lines and two of six mutants in clinical glioblastoma samples. The true and false positive rates were determined at various stringency levels. Importantly, PAC identified a number of novel genetic changes, including those affecting splicing, that previously had gone undetected. These novel genetic changes are either in known cancer genes (*EGFR*), result in a frameshift (*FCGBP*) or are rendered “not tolerated” by gene prediction algorithms PMut and SIFT BLink (*TLE2*). Additional experiments are required to determine whether changes in the novel candidate cancer genes (*FCGBP* and *TLE2*) are indeed oncogenic. A significant number of nucleotide substitutions that are located within the probe set selection region are also PAC detected (Fig. 5). Our results thus classify PAC as a reliable approach to screen for candidate cancer genes in a global fashion.

Gene expression profiling at the level of individual exons has only recently become feasible through the release of exon arrays. Here, we have explored the efficacy of PAC to identify exon-skipping mutants, but the strategy may also be used to deduce the primary structure of gene transcripts [13], [22]. It is important to note that the PAC algorithm, detailed under Materials and Methods, is in essence a simple formula that predicts outlier exons based on fold change differences between measured and predicted exon expression levels. Other approaches can also be used to identify outliers (e.g. >n standard deviations from the mean expression level) but need to account for the non-linearity in gene expression levels between samples (especially for cancer genes) and the limited sample size. Because of the high true positive rates obtained by PAC, we did not further explore alternative statistical approaches.

The PAC algorithm is independent of array platform or organism, allowing application of the PAC strategy in a wide variety of biological systems. Several algorithms for exon-level expression profiling are commercially available, including Stratagene ArrayAssist (www.stratagene.com), Partek Genomics Suite (www.partek.com) and Genomatix Suite (www.genomatix.de). Although each of these software packages is relatively straight-forward, important advantages of PAC are that it allows detection of unique outlier exons without any prior knowledge of the encoding gene or its transcript structure and that it does not require predefined subgroups of samples with differential expression of the outlier exons.

As with any global screening strategy, PAC has its preconditions for detecting outlier exons. First and foremost, identification of outlier exons requires their transcript expression level to be within the linear detection range of the exon array, which is determined by their transcript expression level as well as the hybridization efficiency and specificity of the probe sets involved. The constituency of the test samples is another consideration, particularly when both mutant and wild-type transcripts may be expressed. For example, the breast cancer cell line cohort included two splice site mutants that escaped detection by PAC because each had a second transcript length of major intensity that resulted from cryptic splicing (*BRCA1* c.5396+1G>A in MDA-MB-436 [14] and *p16* c.150+2T>C in MDA-MB-436 (Nagel *et al.*, submitted for publication). Furthermore, PAC detection of the *EGFRvIII* transcript isoform in clinical glioblastomas was determined by the overall expression level of *EGFR* transcripts, that was near the limits of linear detection in all five *EGFRvIII* glioblastomas, but also by the ratio of the *EGFRvIII* isoform versus wild-type *EGFR* transcripts (Fig. 3B). A corollary is that PAC performance may be compromised in detecting an outlier exon when wild-type transcripts represent more than one-fourth of all transcripts of that particular gene, which could be the case in tumor samples with less than 75% neoplastic cells. However, expression levels of mutant and wild-type alleles typically are disproportional to their allele frequency and detection by PAC thus again is determined by the (relative) expression level of the outlier transcript. PAC therefore performs best in the absence of wild-type transcript expression. Homozygous transcripts are predominantly found among tumor suppressor genes, where often one allele is mutated accompanied by loss of the other allele.

The influence of allele ratios was further stressed in our simulations of recurrent outlier detection by PAC: The *EGFRvIII* isoform in GBM67 was detected only once it was present as a unique outlier among 14 samples, whereas it had not been detected in our original PAC screen that included five other *EGFRvIII* expressing glioblastomas (Fig. 4A). However, this sub optimal PAC performance appeared not related to the recurrence of outliers, as recurrent outliers were easily identified among cell lines − even when present in five out of six cell lines (Fig. 4B). The simulation experiments also revealed that two cell lines were sufficient to reliably detect outlier exons and that more than eight cell lines did not further improve PAC performance, whereas for clinical tumor samples ten appeared the minimum but twenty would be preferred (Fig. 4).

How efficient might PAC be in detecting mutations in cancer genomes? From our selection of outlier exons, we identified ∼20% (21/112) SNPs, ∼2% (2/112) novel base changes and ∼2% (2/112) exon skipping events. When including all nucleotide substitutions, the false positive rate in these experiments is ∼76%. By extension, amplification and sequencing 1,763 reactions on a single sample (all outliers at PAC values <−4.0) can be expected to yield as much as 34 novel base changes and 34 exon skipping events. Therefore, our approach can be classified as a highly efficient screening method for candidate cancer genes, especially when compared to random selection of exons. Additional studies should then be performed to determine whether identified changes are causal for the tumor formation and/or progression, for example by screening for additional mutations (e.g. deletions, missense mutations) in other tumor samples or by functional analysis of the identified mutants.

## Materials and Methods

### Samples

Our collection of 41 publicly-available human breast cancer cell lines had been subjected to mutational screens of seven tumor suppressor genes: *BRCA1* (Breast Cancer Susceptibility Gene 1; OMIM 113705), *CDH1* (E-cadherin; OMIM 192090), *MAP2K4* (MAP Kinase Kinase 4, a.k.a. *MKK4*; OMIM 601335), *PTEN* (Phosphatase and Tensin Homolog; OMIM 601728), *p16* (CDK4-inhibitor, a.k.a. *INK4A*, *CDKN2A*; OMIM 600160), *p53* (Tumor Protein p53; OMIM 191170) and *RB1* (Retinoblastoma Susceptibility Gene 1; OMIM 180200) [14]–[18] (Nagel *et al.* submitted for publication). Mutational analysis involved sequencing the entire coding region of these genes on genomic DNA as well as analysis of the encoded transcript. The twelve breast cancer cell lines used for this study were: CAMA-1, EVSA-T, HCC1937, MDA-MB-134VI, MDA-MB-157, MDA-MB-435s, MDA-MB-436, MDA-MB-453, MDA-MB-468, MPE600, OCUB-F and SK-BR-5. Clinical glioblastoma specimens were frozen in liquid nitrogen immediately upon surgical resection from patients at Erasmus University Medical Center, as described elsewhere [13]. Pathological review revealed at least 70% tumor nuclei for each specimen. Mutation analysis of the *EGFR* oncogene (Epidermal Growth Factor Receptor; OMIM 131550) in the glioblastomas was performed by conventional RT-PCR and subsequent sequencing of transcripts from samples with *EGFR* amplifications. *EGFR* transcript expression was quantified by Real-Time RT-PCR, using primers that amplified exons 2–3 or exons 22–23 and thus allowed discrimination of wild-type *EGFR* transcripts and the *EGFRvIII* isoform.

### Exon-level expression profiling

Total RNA was isolated using the Qiagen RNeasy kit for the breast cancer cell lines and using Trizol followed by RNeasy for the glioblastoma specimens [23]. RNA quality was assessed using the Agilent Bioanalyser, requiring RNA integrity >7.0 [24]. All further processing of the samples was performed according the Affymetrix GeneChip Whole Transcript (WT) Sense Target Labeling Assay. Affymetrix GeneChip Human Exon 1.0 ST Arrays were used to determine the expression level of virtually all exons present in the human genome (1.4 million probe sets covering >1 million exon clusters). For this study, we used expression data of the 290,000 core probe sets that are supported by putative full-length mRNA from e.g. the RefSeq database (Geo dataset accession number GSE9385) . Signal processing was performed after sketch normalization by using Affymetrix ExACT 1.2.1 software and the PLIER algorithm, described in Affymetrix GeneChip Exon Array Whitepaper “Gene Signal Estimates from Exon Arrays” and Technote “Guide to Probe Logarithmic Intensity Error (PLIER) Estimation” (www.affymetrix.com/support/technical).

### PAttern based Correlation (PAC)

Predicted exon expression levels were calculated by using the PAC algorithm, described in Whitepaper “Alternative Transcript Analysis Methods for Exon Arrays”, where the predicted expression level of the exon (Exon-pr) equals the overall expression of its transcript in that sample (Transcript-m: the meta probe set expression level) multiplied by the average expression level of that exon among all samples (Exon-ave) and divided by the average overall expression of the transcript among all samples (Transcript-ave), all 2-logarithm transformed. In formula:
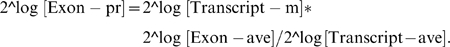



PAC values were calculated by subtracting the predicted expression level of the exon in that sample from its measured expression level (Exon-m), again with 2-logarithm transformation:
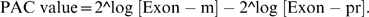



Meta probe set expression levels were calculated using all core probe sets of a transcript with PLIER signal estimates >50. To enrich for probe sets with significant expression above background, PAC values were calculated using exons and transcripts that had PLIER signal estimates >50 [13]. Identification of outlier exons was performed without prior knowledge of the mutation data.

## Supporting Information

Table S1 Complete list of outlier exons at PAC values <−2.0
(13.64 MB XLS)

Table S2 Summary of PCR confirmation results for a randomly selected cohort of outlier exons
(0.04 MB XLS)
